# Intra- and postoperative relative angiotensin II deficiency in patients undergoing elective major abdominal surgery

**DOI:** 10.3389/fendo.2024.1375409

**Published:** 2024-07-08

**Authors:** Katharina Krenn, Petra Höbart, Lukas Adam, Gregor Riemann, Finn Christiansen, Oliver Domenig, Roman Ullrich

**Affiliations:** ^1^ Department of Anesthesia, General Intensive Care and Pain Medicine, Medical University of Vienna, Vienna, Austria; ^2^ Attoquant Diagnostics GmbH, Vienna, Austria; ^3^ Department of Anesthesiology and Intensive Care Medicine, AUVA Trauma Center, Vienna, Austria

**Keywords:** anesthesia, major abdominal surgery, perioperative period, ACE2 (angiotensin converting enzyme 2), renin-angiotensin aldosterone system

## Abstract

**Introduction:**

The classical axis of the renin–angiotensin system (RAS) makes an important contribution to blood pressure regulation under general anesthesia via the vasopressor angiotensin II (Ang II). As part of the alternative RAS, angiotensin-converting enzyme 2 (ACE2) modulates the pro-inflammatory and fibrotic effects of Ang II by processing it into the organ-protective Ang 1–7, which is cleaved to Ang 1–5 by ACE. Although the levels of ACE2 may be associated with postoperative complications, alternative RAS metabolites have never been studied perioperatively. This study was designed to investigate the perioperative kinetics and balance of both RAS axes around major abdominal surgery.

**Methods:**

In this observational cohort study, 35 patients undergoing elective major abdominal surgery were included. Blood sampling was performed before and after induction of anesthesia, at 1 h after skin incision, at the end of surgery, and on postoperative days (POD) 1, 3, and 7. The equilibrium concentrations of Ang I–IV, Ang 1–7, and Ang 1–5 in plasma were quantified using mass spectrometry. The plasma protein levels of ACE and ACE2 were measured with ELISA.

**Results:**

Surgery caused a rapid, transient, and primarily renin-dependent activation of both RAS axes that returned to baseline on POD 1, followed by suppression. After induction, the Ang II/Ang I ratio persistently decreased, while the ACE levels started to increase on POD 1 (all *p* < 0.01 *versus* before anesthesia). Conversely, the ACE2 levels increased on POD 3 and 7 (both *p* < 0.001 *versus* before anesthesia), when the median Ang 1–7 concentrations were unquantifiably low.

**Discussion:**

The postoperative elevation of ACE2 may prolong the decrease of the Ang II/Ang I ratio through the increased processing of Ang II. Further clarification of the intraoperative factors leading to relative Ang II deficiency and the sources of postoperatively elevated ACE2 is warranted.

## 1 Introduction

Anesthesia and surgery induce the activation of the renin–angiotensin system (RAS) (1, 2) since the vasoconstrictor angiotensin II (Ang II) (1), in concert with the sympathetic nervous system (3) and the release of vasopressin (4), prevents the hypotension caused by vasodilation during general anesthesia. Together, these compensatory systems maintain systemic blood pressure even in the case of pharmacological inhibition (1, 5). However, Ang II may also have detrimental pro-inflammatory and fibrotic effects, which are modulated by the angiotensin-converting enzyme 2 (ACE2) and the alternative RAS axis (6, 7). ACE2 processes Ang II to Ang 1–7, which mediates vasodilation and the anti-inflammatory and anti-fibrotic effects through the Mas receptor (6–9) and protects from experimental lung injury (10–12). Ang 1–7 is a substrate of ACE and, in circulation, is rapidly cleaved to Ang 1–5 (13). The balance of the classical and alternative RAS has been studied in chronic heart (14, 15) and kidney (16) diseases, and the increased activation of the alternative RAS, together with a reduced Ang II/Ang I ratio, has been observed in mechanically ventilated patients with acute respiratory distress syndrome (ARDS) caused by various triggers (17, 18), including coronavirus disease 2019 (COVID-19) (19).

Death within 30 days after surgery is one of the most frequent causes of death worldwide (20). Earlier studies have shown that the levels of soluble ACE2 are associated with postoperative complications (21–24). While complications following emergency orthopedic surgery (21) and esophagectomy (24) are associated with higher ACE2 levels, complications after a major pulmonary resection (22) and coronary artery bypass grafting (23) are more frequent in patients with the lowest ACE2 levels. These divergent results could be associated with the comorbidities, the type of surgery, and the analyzed outcomes. However, there still is little knowledge as to whether soluble ACE2 impacts the systemic levels of the effector metabolite Ang 1–7 in the perioperative period and whether the classical and the alternative axes of the RAS are equally activated during surgery.

To better delineate the perioperative RAS response from before anesthesia until 1 week after surgery, we investigated the kinetics of the angiotensin metabolite levels (Ang I–IV, Ang 1–7, and Ang 1–5), the plasma renin activity (PRA), the aldosterone concentrations, the angiotensin metabolite concentration-based markers of RAS enzyme activities, and the ACE and ACE2 protein levels in a prospective study that included patients undergoing major abdominal surgery without previous exposure to RAS-modifying drugs.

## 2 Materials and methods

### 2.1 Study design and setting

This prospective study was assessed favorably by the Ethics Committee of the Medical University of Vienna, Austria (protocol no. 1446/2017). All patients provided written informed consent before inclusion in the study, which was performed at the Department of Anesthesia and General Intensive Care of the Medical University of Vienna between March 2018 and August 2021. The study design and data handling followed the STROBE guidelines for observational cohort studies. Anesthesia and fluid management were handled by the responsible anesthesiologists according to institutional standards. The standard intraoperative crystalloid fluid therapy was Elomel Isoton^®^ (Fresenius Kabi Austria, Vienna, Austria), and, if required, the standard colloid therapy was 20% human albumin.

### 2.2 Inclusion and exclusion criteria

The inclusion criteria were age between 18 and 80 years and elective abdominal surgery or thoraco-abdominal esophagectomy with an expected duration of >2 h that required intraoperative invasive arterial blood pressure monitoring.

Patients were excluded if they had been taking RAS-modifying drugs within the last week or had a history of chronic renal failure with creatinine levels >1.5 mg/dL (corresponding to chronic kidney disease stages 4–5 in patients 18–80 years of age), liver cirrhosis of Child–Pugh grade B or higher, hormone-producing tumors, or if surgery was expected to involve the kidneys or adrenal glands to avoid additional perturbances of the RAS.

### 2.3 Blood sampling

Blood was collected in Li-heparin tubes before and after induction of anesthesia, at 1 h following skin incision, at the end of surgery (EOS) before extubation, and on postoperative days (POD) 1, 3, and 7. After centrifugation for 10 min at 1,500 rpm, plasma was frozen at −40°C until analysis.

### 2.4 RAS equilibrium analysis

*Ex vivo* equilibrium concentrations of the angiotensin peptides [Ang I (Ang 1–10), Ang II (Ang 1–8), Ang III (Ang 2–8), Ang IV (Ang 3–8), Ang 1–7, and Ang 1–5] and the concentrations of aldosterone in plasma were measured using liquid chromatography–mass spectrometry/mass spectroscopy (LC-MS/MS; Attoquant Diagnostics GmbH, Vienna, Austria) as described (14, 25, 26). The lower limits of quantification (LLOQs) were 2 pmol/L for Ang II, Ang 1–5, and Ang IV; 3 pmol/L for Ang 1–7 and Ang III; 4 pmol/L for Ang I; and 10 pmol/L for aldosterone. The aldosterone/Ang II ratio (AA2R) was calculated as described (27).

### 2.5 Angiotensin-based markers of RAS enzyme activities

The activities of the angiotensin-cleaving enzymes in plasma were assessed through calculated markers using the measured angiotensin metabolite concentrations: PRA-S (Ang I + Ang II) as a measure of PRA (26, 27), ACE-S (Ang II/Ang I ratio) (27) and the Ang 1–5/Ang 1–7 ratio (28) as the markers of ACE activity, and ALT-S [(Ang 1–7 + Ang 1–5)/(Ang I + Ang II + Ang 1–7 + Ang 1–5)] as a marker of alternative RAS activation (11).

### 2.6 Renin activity assay

PRA was measured using an LC-MS/MS-based Ang I generation assay (27) as an intrinsic Ang I formation rate (in nanograms Ang I per milliliter per hour) with an LLOQ of 0.02 ng Ang I mL^−1^ h^−1^.

### 2.7 ELISA

The protein levels of ACE and ACE2 in plasma were measured with ELISA according to the manufacturer’s protocol (Cloud Clone Corp., Katy, TX, USA).

### 2.8 Statistics

IBM SPSS Statistics 28.0.1.0 and GraphPad Prism 9.1.1 were used for data analysis. As the primary outcome, the RAS parameters along the perioperative timeline were compared to those of the baseline (before anesthesia) using mixed-effects analysis with Dunnett’s correction, while the Benjamini–Hochberg (BH) procedure (29) was applied to correct for multiple testing assuming a false discovery rate of 5%. Only *p*-values less than the BH critical value were considered significant. The sample size for the primary outcome was based on the statistically significant differences found in the Ang II levels before and after induction of anesthesia in 27 patients (2) and the postoperative return of the Ang II levels to baseline in another study including 17 patients (30).

All other secondary hypotheses were tested in exploratory analyses without adjustment for multiple testing, and *p*-values <0.05 were considered significant. Correlations between the clinical parameters and the RAS parameters were analyzed using Spearman’s rank test. Differences between groups were analyzed with the Mann–Whiney *U* test. The association between PRA and PRA-S was analyzed with linear regression analysis. The associations between the Ang 1–7 and Ang 1–5 concentrations (dependent variables) and PRA, the aldosterone concentration, ACE-S, the ACE2 levels, systolic blood pressure, and peak inspiratory pressure (independent variables) were analyzed using multivariate linear regression models with stepwise backward elimination at “1 h after skin incision” and “EOS.” The mixed-effects analyses, correlations, and linear regression analyses were performed using log_10_-transformed RAS data to eliminate the effect of non-normal distributions. For concentrations below the LLOQ, the respective LLOQ divided by the square root of 2 was used for calculations (31).

## 3 Results

### 3.1 Study population and clinical parameters in the perioperative period

**Figure 1:**
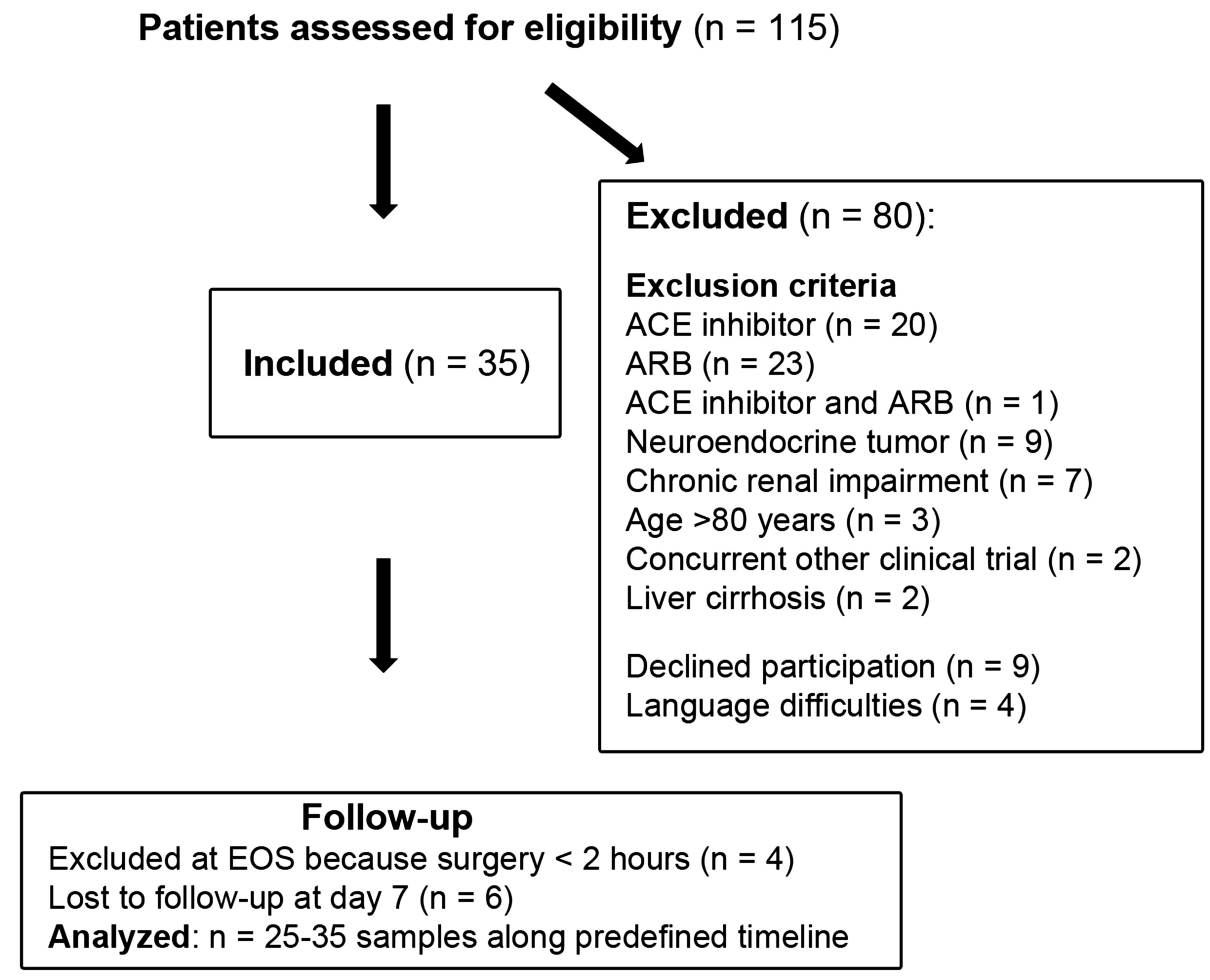
Flowchart of the course of the study from enrollment to data analysis.

Within the study period, 115 patients undergoing elective major abdominal surgery were screened for participation. A total of 35 patients were enrolled (**Figure 1**). The most common exclusion criterion was treatment with RAS-modifying drugs (*n* = 44). The mean age of the included patients was 54 ± 14 years (range, 29–77 years), and 57% were women. The demographics, chronic comorbidities, and types of surgery are shown in **Table 1**.

**Table 1:** Baseline and perioperative characteristics of the study participants.

Parameter	Patients (*n* = 35)
Age (years)	54 ± 14
Gender (M/F), *n* (%)	15/20 (43/57)
BMI (kg/m^2^)	25.2 ± 5.0
Chronic comorbidities (with *n* ≥ 4), *n* (%)	
Cancer (reason for surgery)	28 (80)
Arterial hypertension	7 (20)
Diabetes mellitus type II	6 (17)
Pulmonary disease (COPD, asthma)	6 (17)
Thyroid disease	4 (11)
Laboratory parameters	
Preoperative creatinine (mg/dL)	0.79 (0.62–0.91)
Preoperative bilirubin (mg/dL)	0.40 (0.23–0.68)
Type of surgery, *n* (%)	
Colorectal	11 (31)
Pancreatic	6 (17)
Gastrectomy	4 (11)
CRS+HIPEC	4 (11)
Other	10 (29)
Duration of surgery (min)	261 ± 145
Thoracic epidural anesthesia, *n* (%)	15 (57)
Intraoperative corticosteroids, *n* (%)	17 (49)
Intraoperative noradrenaline, *n* (%)	27 (77)
Intraoperative cumulative noradrenaline dose (mg)	0.35 (0.05–1.35)
Intraoperative phenylephrine, *n* (%)	28 (80)
Intraoperative cumulative phenylephrine dose (mg)	0.26 (0.06–0.46)
Intraoperative fluid balance (mL)	+2,350 (1,410–3,190)
Postoperative mechanical ventilation, *n* (%)	5 (14)
Length of hospital stay (days)	10 (8–12)

Data are expressed as numbers and percentages, mean ± SD, or median (25th–75th percentile) according to the scale and distribution of the variable.

BMI, body mass index; COPD, chronic obstructive pulmonary disease; CRS, cytoreductive surgery; HIPEC, hyperthermic intraperitoneal chemotherapy.

After induction of anesthesia with intravenous fentanyl, propofol (or etomidate in one case), and rocuronium, general anesthesia was maintained with sevoflurane and fentanyl or sufentanil. The time elapsed between the propofol injection and blood sampling after induction was 2–30 min (9 ± 6 min). In 15 (43%) patients, a thoracic epidural catheter was placed after the baseline sample was taken and before induction of general anesthesia. A total of 17 (49%) patients received a single dose of a corticosteroid after induction to prevent postoperative nausea and vomiting (*n* = 16) or swelling after difficult intubation (*n* = 1). There were four patients excluded from follow-up as the intended surgery was aborted without resection due to peritoneal carcinosis, with duration of <2 h (**Figure 1**).

The drop in the mean arterial blood pressure (MAP) after induction of anesthesia was counterbalanced with bolus doses of intravenous phenylephrine in 28 of 35 (80%) patients (**Table 1**). The hemodynamic and ventilation parameters remained stable during surgery (**Table 2**). The majority (*n* = 27, 77%) of patients received a continuous infusion of noradrenaline during surgery (**Table 1**).

**Table 2:** Hemodynamic and respiratory parameters of the study participants in the operating room.

Parameter	Baseline	ANE	1 h of surgery	EOS
BP systolic (mmHg)	138 ± 21	98 ± 17	108 ± 17	106 ± 16
BP diastolic (mmHg)	79 ± 11	61 ± 10	59 ± 8	57 ± 9
BP mean (mmHg)	104 ± 15	75 ± 12	77 ± 10	75 ± 10
Heart rate (min^−1^)	73 ± 12	72 ± 15	68 ± 11	70 ± 13
PPeak (mbar)	–	15 (13–18)	15 (13–17)	16 (14–17)
PDr (mbar)	–	9 (8–12)	9 (9–11)	10 (9–12)
PEEP (mbar)	–	5 (5–6)	5 (5–6)	5 (5–6)
Respiratory rate (min^−1^)	–	12 (12–12)	12 (12–13)	13 (12–14)
PaO_2_/FiO_2_ (mmHg)	–	456 (364–538)	426 (320–483)	434 (372–485)
PaCO_2_ (mmHg)	–	40.5 (37.8–45.8)	42.1 (39.7–45.6)	40.9 (39.3–43.2)
VT (mL/kg PBW)	–	7.5 (6.5–9.3)	7.6 (7.3–8.4)	7.5 (7.1–8.5)
Compliance (mL/mbar)	–	54.9 ± 14.8	52.1 ± 10.5	49.7 ± 10.7

Data are shown as the mean ± SD or median (25th–75th percentile). Compliance = VT/PDr.

ANE, after induction of anesthesia; 1 h of surgery, 1 h after skin incision; EOS, end of surgery; BP, blood pressure; PPeak, peak inspiratory pressure; PDr, driving pressure; PEEP, positive end-expiratory pressure; VT, tidal volume; PBW, predicted body weight.

In the patients who received thoracic epidural anesthesia, the MAP was lower after induction of general anesthesia (*p* = 0.030), and the cumulative intraoperative noradrenaline dose was higher (*p* = 0.030) than that in patients without epidural anesthesia. The median fluid balance at EOS was +2,350 mL (1,410–3,190 mL) (**Table 1**). There were five patients who were mechanically ventilated and sedated when transferred to the intensive care unit (**Table 1**), and all of them were extubated until the next morning. The postoperative clinical parameters are summarized in **Table 3**. Routine postoperative laboratory showed increased C-reactive protein (CRP) levels and leukocyte counts and, in one case, acute kidney injury of KDIGO grade 1 (32). The median length of hospital stay (LOS) was 10 days (8–12 days) (**Table 1**). Postoperative complications occurred in five patients, i.e., intestinal anastomosis insufficiency, postoperative bleeding, a fascial dehiscence of the laparotomy, an allergic reaction, and one case of sepsis.

**Table 3:** Postoperative clinical parameters of the patients after major abdominal surgery.

Parameter	POD 1	POD 3	POD 7
BP systolic (mmHg)	115 ± 21	128 ± 17	125 ± 21
BP diastolic (mmHg)	58 ± 12	72 ± 14	68 ± 10
BP mean (mmHg)	78 ± 14	91 ± 14	87 ± 13
Heart rate (min^−1^)	73 ± 13	82 ± 11	79 ± 8
Fluid balance (mL)	+823 (389–1286)	–	–
Creatinine (mg/dL)	0.76 (0.63–0.98)	–	–
Bilirubin (mg/dL)	0.51 (0.34–0.82)	–	–
CRP (mg/dL)	6.53 (3.69–9.80)	7.53 (4.02–17.61)	4.93 (3.07–7.82)
Leukocyte count (G/L)	11.05 (7.96–13.57)	8.79 (6.35–11.31)	8.96 (5.79–12.42)

Data are shown as the mean ± SD or median (25th–75th percentile).

POD, postoperative day; BP, blood pressure; CRP, C-reactive protein.

### 3.2 Perioperative activation of the classical and alternative RAS

**Figure 2:**
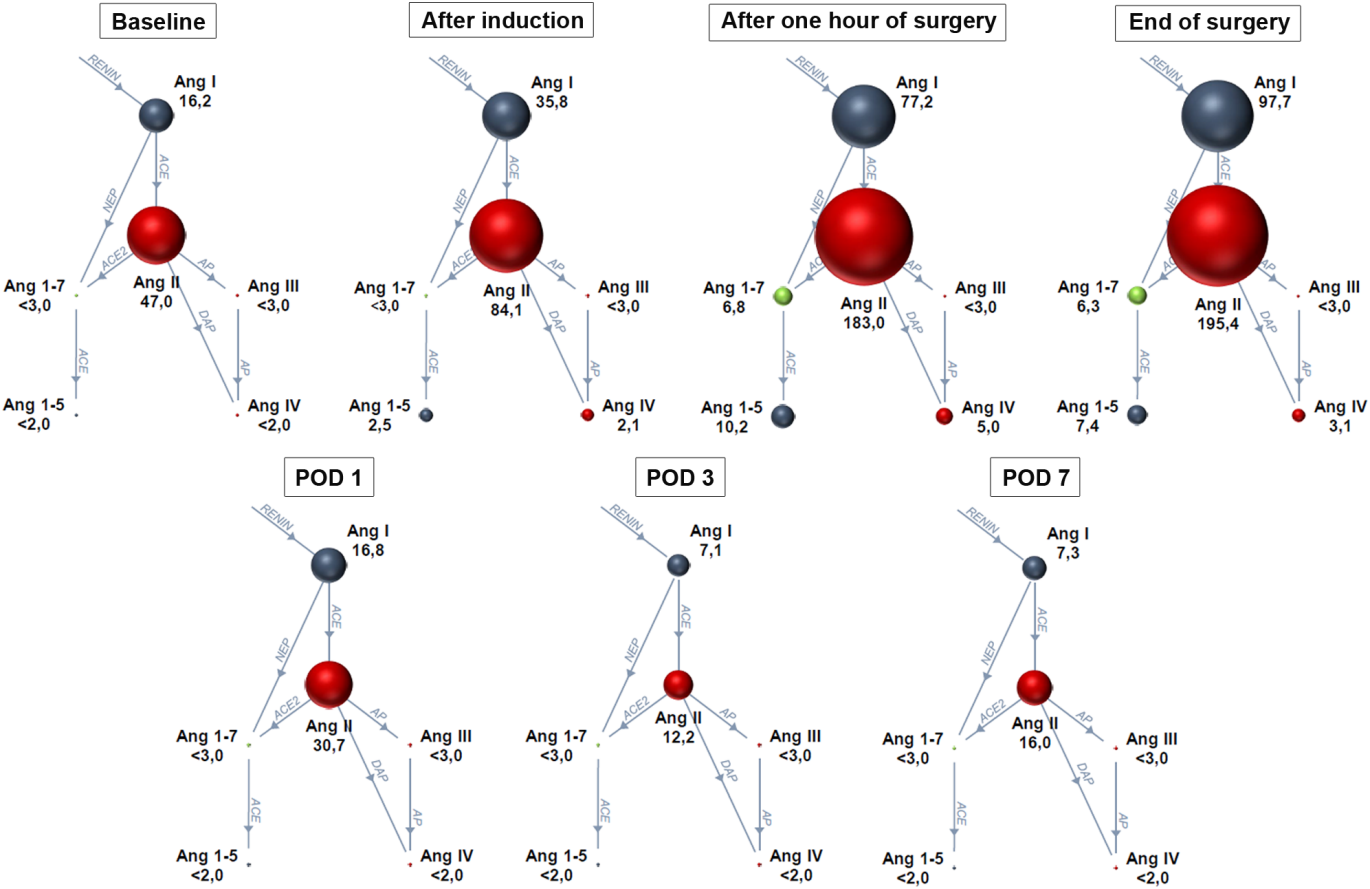
Renin–angiotensin system fingerprints along the predefined perioperative timeline. *Baseline*: before anesthesia (*n* = 25), *after induction* of anesthesia (*n* = 35), *after 1 h of surgery* (*n* = 35), at the *end of surgery* (*n* = 31), and on postoperative days (*POD*) *1* (*n* = 31), *3* (*n* = 31), and *7* (*n* = 25). *Sphere sizes* correspond to the median angiotensin metabolite concentrations in plasma. *Ang*, angiotensin; *ACE*, angiotensin-converting enzyme; *NEP*, neprilysin; *AP*, aminopeptidase; *DAP*, dipeptidyl aminopeptidase. Data are presented as medians. <2 or <3 indicates median value below the lower limit of quantification.

**Figure 3:**
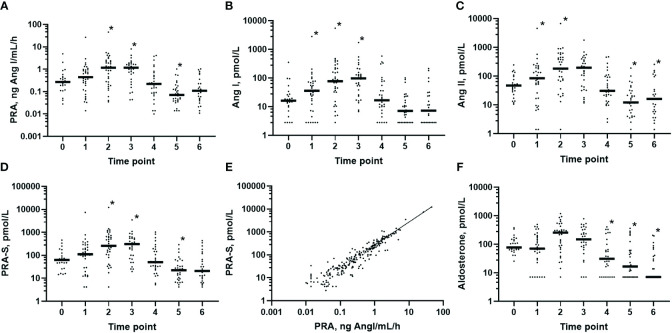
Pattern of the classical renin–angiotensin aldosterone system activation in plasma in the perioperative period. Predefined points in time for sampling: *0*, before anesthesia (*n* = 25); *1*, after induction (*n* = 35); *2*, 1 h after skin incision (*n* = 35); *3*, end of surgery (*n* = 31); *4*, postoperative day 1 (*n* = 31); *5*, postoperative day 3 (*n* = 31); and *6*, postoperative day 7 (*n* = 25). **(A)** PRA (plasma renin activity): 0 *vs*. 2, 3, and 5 (*p* < 0.001). **(B)** Angiotensin I (Ang I) concentrations: 0 *vs*. 1 (*p* = 0.002) and 0 *vs*. 2 and 3 (*p* < 0.001). **(C)** Ang II concentrations: 0 *vs*. 2, 3, and 5 (*p* < 0.001) and 0 *vs*. 6 (*p* = 0.01). **(D)** PRA-S (Ang I + Ang II) as the angiotensin-based marker of renin activity: 0 *vs*. 2, 3, and 5 (*p* < 0.001). **(E)** Close correlation of PRA-S with PRA (*r* ^2 =^ 0.979, *p* < 0.001). **(F)** Aldosterone concentrations: 0 *vs*. 4 (*p* = 0.004) and 0 *vs*. 5 and 6 (*p* < 0.001). Comparisons of values at baseline = 0 *versus* occasions 1–6 were examined using mixed-effects analysis with Dunnett’s correction. In (**A–D**, **F**), only the *p*-values less than the Benjamini–Hochberg critical value are shown as significant (*asterisk*).

The RAS fingerprints illustrating the course of perioperative RAS activation are shown in **Figure 2**, and the median values of all RAS parameters are summarized in **Table 4**. Ang I increased after induction of anesthesia (*p* = 0.002 *versus* baseline before anesthesia), and PRA, Ang I, Ang II, and the renin activity marker PRA-S reached peak values during surgery (all *p* < 0.001 after 1 h of surgery *versus* baseline) (**Figures 3A–D**). The marker PRA-S strongly correlated with PRA in the linear regression analysis (*r* ^2 =^ 0.979, *p* < 0.001) (**Figure 3E**). The plasma concentrations of aldosterone were unchanged after 1 h of surgery (**Figure 3F**), and the AA2R was reduced *versus* baseline (*p* = 0.005) (**Table 4**). At EOS, the RAS remained activated, as demonstrated by the increased PRA, PRA-S, Ang I, and Ang II concentrations (all *p* < 0.001) (**Figures 3A–D**) compared with those at baseline, while the aldosterone levels remained unchanged and the AA2R remained decreased (*p* = 0.003) (**Table 4**). On POD 1, the markers of classical RAS activation returned to baseline, and the aldosterone concentrations were markedly downregulated (*p* = 0.004) (**Figure 3F**). On POD 3, PRA, the Ang II concentrations, and PRA-S (**Figures 3A, C, D**, respectively) decreased below baseline (all *p* < 0.001), and the aldosterone concentrations remained downregulated (*p* < 0.001) (**Figure 3F**). The Ang II (*p* = 0.009) and aldosterone (*p* < 0.001) concentrations also stayed below baseline on POD 7 (**Figures 3C, F**) in the patients still hospitalized (*n* = 25).

**Table 4:** Renin–angiotensin aldosterone system kinetics in patients undergoing major abdominal surgery.

Parameter	Baseline (*n* = 25)	ANE (*n* = 35)	1 hour (*n* = 35)	EOS (*n* = 31)	POD 1 (*n* = 31)	POD 3 (*n* = 31)	POD 7 (*n* = 25)
LC-MS/MS							
PRA (ng Ang I ^−1^ mL h^−1^)	0.270 (0.153–0.440)	0.442 (0.120–0.941)	1.170* (0.269–2.379)	1.169* (0.357–1.696)	0.221 (0.086–0.534)	0.071* (0.039–0.149)	0.108 (0.042–0.292)
Aldosterone (pmol/L)	77.6 (63.3–147.9)	70.3 (28.6–185.2)	255.8 (77.6–426.8)	148.0 (77.5–334.7)	31.1* (<10–99.6)	16.6* (<10–60.9)	<10* (<10–54.4)
Ang I (pmol/L)	16.2 (8.5–26.8)	35.8* (7.3–72.6)	77.2* (32.1–217.0)	97.7* (25.9–156.6)	16.8 (7.5–75.6)	7.1 (<4–22.2)	7.3 (<4–27.6)
Ang II (pmol/L)	47.0 (25.8–86.7)	84.1 (17.0–194.6)	183.0* (71.9–449.3)	195.4* (41.7–365.4)	30.7 (13.5–97.3)	12.2* (5.3–30.7)	16.0* (5.4–64.8)
Ang III (pmol/L)	<3 (<3 to <3)	<3 (<3 to <3)	<3* (<3–7.5)	<3 (<3–5.0)	<3 (<3 to <3)	<3 (<3 to <3)	<3 (<3 to <3)
Ang IV (pmol/L)	<2 (<2 to <2)	2.1* (<2–5.5)	5.0* (<2–12.3)	3.1* (<2–7.4)	<2 (<2–3.4)	<2 (<2 to <2)	<2 (<2 to <2)
Ang 1–7 (pmol/L)	<3 (<3 to <3)	<3 (<3–3.5)	6.8* (<3–15.2)	6.3* (<3–15.5)	<3 (<3–6.7)	<3 (<3 to <3)	<3 (<3 to <3)
Ang 1–5 (pmol/L)	<2 (<2–4.0)	2.5 (<2–6.3)	10.2* (<2–19.7)	7.4* (<2–)12.3	<2 (<2–)5.2	<2 (<2 to)<2	<2 (<2–)3.9
PRA-S (pmol/L)	63.0 (36.8–109.6)	111.6 (28.8–284.1)	259.3* (104.0–673.3)	308.0* (72.4–554.1)	50.3 (25.9–185.5)	22.5* (9.2–57.7)	21.1 (8.7–92.9)
ACE-S	3.3 (2.3–4.2)	2.7* (1.4–3.6)	2.4 (1.9–3.3)	1.9* (1.0–2.7)	2.0* (1.2–2.7)	1.4* (1.1.–2.4)	1.4* (1.0–2.9)
ALT-S	0.05 (0.04–0.08)	0.04 (0.04–0.06)	0.05 (0.03–0.06)	0.05 (0.03–0.07)	0.08 (0.06–0.12)	0.14 (0.07–0.19)	0.07 (0.04–0.13)
AA2R	2.13 (1.35–3.44)	0.90 (0.42–2.57)	1.16* (0.53–1.75)	0.92* (0.46–2.49)	1.22 (0.56–1.65)	1.64 (0.81–2.86)	1.04 (0.68–3.42)
ELISA							
ACE (ng/mL)	455.2 (247.8–651.8)	604.7 (444.7–677.1)	533.5 (400.4–619.9)	628.4 (361.1–734.7)	691.7* (554.1–892.0)	687.2* (549.5–848.2)	768.3* (637.4–983.6)
ACE2 (pg/mL)	989.8 (446.4–1,828.7)	1,342.3 (420.4–2,084.4)	1,423.9 (767.7–3,015.3)	2,154.2 (1,225.3–3,193.4)	2,117.4 (1,202.5–3,340.3)	3,860.6* (2,336.6–7,081.5)	4,624.6* (1,795.0–7,991.2)

Data are shown as the median (25th–75th percentile). Comparisons of values at baseline versus occasions ANE to POD 7 were examined using mixed-effects analysis with Dunnett’s correction. Asterisk denotes p-values less than the Benjamini–Hochberg critical value, which are indicated as significant.

Baseline, before anesthesia; ANE, after induction; 1 hour, 1 h after skin incision; EOS, end of surgery; POD, postoperative day; LC-MS/MS, Liquid chromatography–tandem mass spectrometry; PRA, plasma renin activity; Ang, angiotensin; PRA-S, Ang I + Ang II; ACE-S, Ang II/Ang I; ALT-S, [(Ang 1–7 + Ang 1–5)/(Ang I + Ang II + Ang 1–7 + Ang 1–5)]; AA2R, aldosterone/Ang II ratio; ACE, angiotensin-converting enzyme.

**Figure 4:**
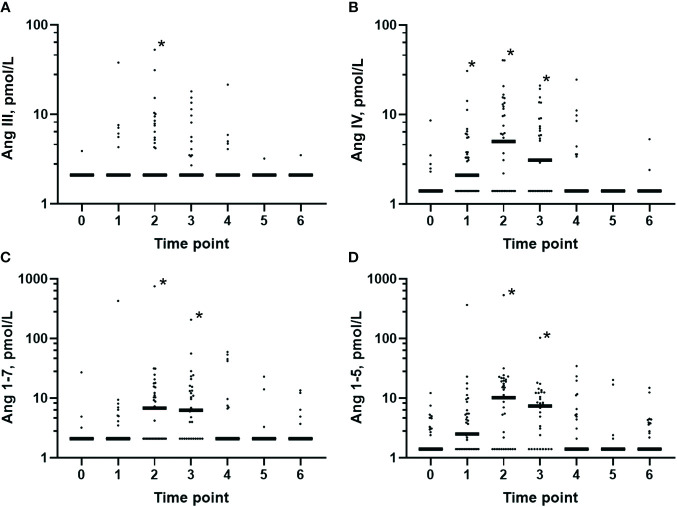
Plasma levels of the smaller angiotensin metabolites in the perioperative period. Predefined timeline for sampling: *0*, before anesthesia (*n* = 25); *1*, after induction (*n* = 35); *2*, 1 h after skin incision (*n* = 35); *3*, end of surgery (*n* = 31); *4*, postoperative day 1 (*n* = 31); *5*, postoperative day 3 (*n* = 31); and *6*, postoperative day 7 (*n* = 25). The plasma concentrations of all of these smaller angiotensin metabolites were increased 1 h after skin incision and at the end of surgery *versus* baseline before anesthesia. Classical renin–angiotensin system (RAS) axis: **(A)** Angiotensin (Ang) III: 0 *vs*. 2 (*p* = 0.01). **(B)** Ang IV: 0 *vs*. 1 (*p* = 0.01), 0 *vs*. 2 (*p* < 0.001), and 0 *vs*. 3 (*p* = 0.002). Alternative RAS axis: **(C)** Ang 1–7: 0 *vs*. 2 and 3 (*p* < 0.001). **(D)** Ang 1–5: 0 *vs*. 2 and 3 (*p* < 0.001). Comparisons of the values at baseline = 0 *versus* occasions 1–6 were assessed using mixed-effects analysis with Dunnett’s correction. Only *p*-values less than the Benjamini–Hochberg critical value are shown as significant (*asterisk*).

Before induction of anesthesia, after 1 h of surgery, and at EOS, Ang III and Ang IV were detectable in plasma in 4%, in 43% and 42%, and in 20%, 57%, and 55% of patients, respectively. After 1 h of surgery, the Ang III and Ang IV levels were higher than those at baseline (*p* = 0.007 and *p* = 0.002, respectively) (**Figures 4A, B**), while the Ang IV levels remained elevated at EOS (*p* = 0.002). In the postoperative period, the Ang III and Ang IV levels were only rarely detectable.

Before induction of anesthesia, at 1 h after skin incision, and at EOS, Ang 1–7 and Ang 1–5 were detectable in 12%, in 54% and 65%, and in 44%, 69%, and 71% of patients, respectively. At 1 h after skin incision and at EOS, the Ang 1–7 and Ang 1–5 concentrations were higher than those at baseline (both *p* < 0.001) (**Figures 4C, D**) and decreased below the LLOQ in the postoperative period in the majority of patients.

### 3.3 Perioperative ACE and ACE2 concentrations and markers for RAS enzyme activities

**Figure 5:**
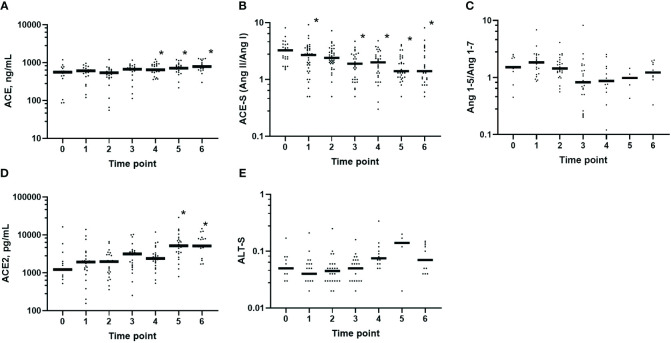
Perioperative protein levels and activity of the angiotensin-processing enzymes in plasma. Predefined timeline for sampling: *0*, before anesthesia (*n* = 25); *1*, after induction (*n* = 35); *2*, 1 h after skin incision (*n* = 35); *3*, end of surgery (*n* = 31); *4*, postoperative day 1 (*n* = 31); *5*, postoperative day 3 (*n* = 31); and *6*, postoperative day 7 (*n* = 25). The protein levels of the angiotensin-converting enzyme (ACE) and ACE2 increased postoperatively, while ACE-S declined starting after the induction of anesthesia. **(A)** ACE protein levels: 0 *vs*. 4 (*p* = 0.002), 0 *vs*. 5 (*p* < 0.001), and 0 *vs*. 6 (*p* = 0.001). **(B)** ACE-S (Ang II/Ang I) as a marker of ACE activity: 0 *vs*. 1 (*p* = 0.01), 0 *vs*. 3 (*p* = 0.001), 0 *vs*. 4 (*p* = 0.003), and 0 *vs*. 5 and 6 (*p* < 0.001). **(C)** Ang 1–5/Ang 1–7 ratio as a marker of ACE N-domain activity, with no significant differences along the timeline. **(D)** ACE2 protein levels: 0 *vs*. 5 and 6 (*p* < 0.001). **(E)** ALT-S [(Ang 1–7 + Ang 1–5)/(Ang I + Ang II + Ang 1–7 + Ang 1–5)] as a marker of alternative RAS activation, with no significant differences along the timeline. Comparisons of the values at baseline = 0 *versus* occasions 1–6 were examined using mixed-effects analysis with Dunnett’s correction. Only *p*-values less than the Benjamini–Hochberg critical value are shown as significant (*asterisk*).

On POD 1, 3, and 7, the protein levels of ACE in plasma showed a persistent increase compared with those at baseline (*p* = 0.002, *p* < 0.001, and *p* = 0.001, respectively) (**Figure 5A**). This was in sharp contrast to the reduced ACE-S (Ang II/Ang I ratio) (**Figure 5B**) that declined after the induction of anesthesia (*p* = 0.008) and remained below the baseline until POD 7 (*p* = 0.001 at EOS, *p* = 0.003 on POD 1, and *p* < 0.001 on POD 3 and 7). However, the Ang 1–5/Ang 1–7 ratio, a measure of ACE N-domain activity, remained unchanged until POD 7 (**Figure 5C**), which could indicate that not only the reduced ACE activity but also the increased Ang II processing by other enzymes led to this reduction in ACE-S.

The protein levels of ACE2 in plasma (**Figure 5D**) started to increase at EOS and were significantly increased on POD 3 and 7 (both *p* < 0.001 *versus* baseline). ALT-S, an angiotensin concentration-based marker of alternative RAS activation (**Figure 5E**), slightly increased in the postoperative period. However, ALT-S and the Ang 1–5/Ang 1–7 ratio could only be calculated in a very low number of patients (POD 3: *n* = 5; POD 7: *n* = 11), provided that at least Ang 1–7 or Ang 1–5 must be detectable above the LLOQ. The protein levels of ACE2 correlated with ALT-S at 1 h after skin incision (*r* = 0.475, *p* = 0.035) and on POD 1 (*r* = 0.627, *p* = 0.039).

### 3.4 Perioperative alternative angiotensin metabolite Ang 1–7 and Ang 1–5 plasma levels are renin-dependent

To determine which factors mainly contributed to the observed increase in the levels of Ang 1–7 and Ang 1–5 during surgery, multivariate linear regression models with stepwise backward elimination were applied. The RAS parameters with a different behavior over time (PRA, aldosterone, ACE-S, and the ACE2 protein levels) were selected, as well as the systolic blood pressure and the peak inspiratory pressure as clinical parameters. In these models, PRA was the most important predictor of the Ang 1–7 and Ang 1–5 concentrations at 1 h after skin incision and at EOS (*p* < 0.001).

### 3.5 Associations between RAS and the clinical parameters

There were no associations between the RAS parameters and the intraoperative peak inspiratory pressure or fluid balance. Higher cumulative intraoperative noradrenalin doses were associated with higher Ang 1–7 concentrations after induction (*r* = 0.368, *p* = 0.029), as well as reduced ACE-S (*r* = −0.442, *p* = 0.008) and increased ALT-S (*r* = 0.529, *p* = 0.008), at 1 h after skin incision, and increased ALT-S on POD 1 (*r* = 0.764, *p* = 0.004).

Epidural anesthesia was associated with lower values of PRA, PRA-S, Ang II, and Ang 1–5 at 1 h after skin incision (all *p* < 0.05) and PRA, Ang 1–5, Ang III, and Ang IV at EOS (all *p* < 0.05). Patients who received a single dose of corticosteroids after induction of anesthesia had increased levels of PRA, PRA-S, Ang I, Ang II, Ang 1–7, and Ang IV only after 1 h of surgery (PRA and Ang I: *p* = 0.007; Ang II, PRA-S, Ang 1–7, and Ang IV: all *p* < 0.05), and the aldosterone levels in these patients were higher at all postoperative measurements *versus* those in patients with no corticosteroid application (all *p* < 0.05).

The levels of ACE-S and Ang IV at EOS correlated indirectly with the duration of surgery (*r* = −0.454, *p* = 0.010, and *r* = −0.363, *p* = 0.045, respectively).

On POD 1, the PRA, PRA-S, Ang I, Ang II, and Ang IV concentrations correlated with the CRP levels (PRA: *r* = 0.467, *p* = 0.009; PRA-S: *r* = 0.433, *p* = 0.017; Ang I: *r* = 0.382, *p* = 0.037; Ang II: *r* = 0.470, *p* = 0.009; and Ang IV: *r* = 0.410, *p* = 0.024), while only PRA slightly correlated with the leukocyte counts (*r* = 0.363, *p* = 0.048). The ACE2 protein levels correlated with the leukocyte counts on POD 1, 3, and 7 (*r* = 0.516, *p* = 0.004; *r* = 0.618, *p* = 0.001; and *r* = 0.459, *p* = 0.036, respectively) and the CRP levels on POD 3 (*r* = 0.575, *p* = 0.002). The aldosterone concentrations were indirectly correlated with the leukocyte counts on POD 7 (*r* = −0.639, *p* = 0.036).

## 4 Discussion

This study shows that general anesthesia and major abdominal surgery lead to a strong activation of the RAS that is propagated into the alternative axis in a renin-dependent manner. These effects peaked after 1 h of surgery and at the end of the operation. Interestingly, a decreasing Ang II/Ang I ratio (ACE-S) was observed starting with the induction of anesthesia, while the ACE and ACE2 protein levels increased postoperatively and the Ang 1–5/Ang 1–7 ratio as a marker of ACE activity remained unchanged. PRA, Ang II, and aldosterone reached their nadir on POD 3 or 7, and concomitantly, the smaller angiotensin metabolites mostly disappeared from circulation. Therefore, a putative effect of the postoperatively elevated ACE2 levels on the formation of Ang 1–7 could not be shown directly. Furthermore, our findings confirm the high degree of correlation between PRA and the angiotensin metabolite concentration-based marker PRA-S (18, 27, 33).

The activation of the RAS by anesthesia has been attributed to the relative intravascular volume depletion induced by pharmacological vasodilation (1). While earlier clinical and experimental studies mainly focused on renin (34) and Ang II (2, 30, 35) during general anesthesia and mechanical ventilation (36, 37), these investigations neither covered the dynamic behavior of the biologically active smaller downstream angiotensin metabolites (6, 38) nor the respective role of the RAS enzymes. Only the levels of ACE2 have been studied as a marker for postoperative complications (21–24). The activation of the alternative RAS axis is important for balancing the increased Ang II levels that occur in various pathologies (6). Ang 1–7 and Ang 1–5 appear in systemic circulation in a large proportion of sedated and mechanically ventilated patients with ARDS (18), possibly as a protective response to these stressors. This activation of the alternative RAS axis is even more pronounced in patients with ARDS due to COVID-19 (19, 39). In this study, anesthesia and surgery highly increased PRA, PRA-S, and the downstream angiotensin metabolite concentrations, confirming for the first time the synchronously increased classical and alternative RAS activity in this setting. Interestingly, the increase in Ang II was more prominent than the increase in aldosterone, leading to reduced AA2Rs during surgery and at EOS.

Another major finding of this study was the marked relative Ang II deficiency (reduced ACE-S) during the entire perioperative period starting with the induction of anesthesia. Also in patients with ARDS (18) and severe COVID-19 (19), the protein levels of ACE-S and ACE behaved differently, and higher levels of Ang I than Ang II have been reported in patients with ARDS (17) and vasodilatory shock (40) as indicators of mortality. In our study, these findings suggest that prolonged mechanical ventilation or surgical stress could induce endogenous ACE inhibition or increased cleavage of Ang II, as there was an inverse correlation between ACE-S at EOS and duration of surgery. One mechanism for the inhibition of endogenous ACE in this study may have been the rapidly increasing intraoperative Ang II levels, which competitively inhibit the C-domain of ACE (41). At the same time, the unchanged Ang 1–5/Ang 1–7 ratios indicate unimpaired activity of the ACE N-domain (28). Another reason for the observed decrease in ACE-S could be the processing of Ang II by dipeptidyl peptidase 3 (DPP3) or ACE2. As a cytosolic enzyme, DPP3 is released upon cell injury (42) and is increased transiently after cardiac surgery (43). Therefore, one explanation for the inverse correlation between ACE-S at EOS and duration of surgery could be the increased processing of Ang II by DPP3 that was released during prolonged surgical trauma. DPP3 is also increased in critically ill patients with COVID-19 (44) and in shock (45) and is associated with worse outcomes. However, being acute intraoperative effects, neither ACE inhibition by the rapidly increasing Ang II levels nor the processing of Ang II by DPP3 explains ACE-S remaining decreased postoperatively. Following cardiac surgery, the levels of DPP3 decreased on the first two PODs (43), meaning that Ang II processing by DPP3 should also have abated on the third POD in our study. Another explanation for the persistent decrease in ACE-S could be the increasing levels of ACE2 on POD 3 and 7. However, the activity of ACE2 could not be reliably assessed based on the concentrations of Ang 1–7 due to the overall RAS suppression on these days. If Ang 1–7 or Ang 1–5 were detectable in plasma, there were also increases in alternative RAS activation (ALT-S) in the postoperative period and a weak correlation between the ACE2 protein levels and ALT-S. The source of the postoperatively increased ACE2 levels is yet unknown and might reflect ACE2 shedding from tissues (46). The potential triggers for ACE2 shedding include inflammation (47), as also suggested by the correlations with the leukocyte count and CRP levels in our study, or perioperative cardiovascular stress (21, 46).

In patients with thoracic epidural anesthesia, the activation of RAS was attenuated at 1 h after skin incision and at EOS, which supports previous findings of suppressed renin release under epidural anesthesia (48).

This study has several limitations, primarily the small sample size and the exploratory analysis of secondary endpoints that precluded conclusive assessment of clinical outcomes. It remains unclear to which extent undiagnosed or untreated arterial hypertension, the relative overrepresentation of women in this study, the supine position before anesthesia, or the preoperative fasting and bowel preparation may have influenced the baseline RAS profile. There were seven patients (20%) with arterial hypertension, which could be one reason for the lower baseline Ang II levels in our study population, as compared with normotensive (49) and healthy individuals (19). We excluded all patients with prior exposure to RAS blockers, but the role of other concomitant medications cannot be addressed due to the small sample size. In addition, this study primarily focused on the equilibrium concentrations of the angiotensin metabolites and the enzyme activities related to ACE and ACE2. The relative impact of aminopeptidases, such as aminopeptidase A (APA) and M (APM), which have been implicated in the regulation of angiotensins (50), was not assessed.

In summary, this study shows that general anesthesia and surgery lead to a pronounced renin-dependent activation of the classical and alternative RAS, which returns to baseline on the first POD, followed by a period of relative suppression. This was accompanied by a relative Ang II deficiency (reduced ACE-S) that lasted into the postoperative period despite the increased ACE protein levels and the unchanged ACE N-domain activity. Its potential reasons (i.e., inhibition of endogenous ACE or the cleavage of Ang II by DPP3 or ACE2), the tissue sources of ACE2, and the potential of ACE2 to predict complications following major abdominal surgery merit further study. Better knowledge about perioperative RAS activity may be clinically relevant to identify the indications for the use of Ang II as a vasopressor or may inform the choice of antihypertensive agents in the first week following major abdominal surgery.

## Data availability statement

The raw data supporting the conclusions of this article will be made available by the authors, without undue reservation.

## Ethics statement

The studies involving humans were approved by Ethics Committee of the Medical University of Vienna. The studies were conducted in accordance with the local legislation and institutional requirements. The participants provided their written informed consent to participate in this study.

## Author contributions

KK: Conceptualization, Data curation, Formal analysis, Investigation, Project administration, Visualization, Writing – original draft. PH: Data curation, Formal analysis, Investigation, Writing – review & editing. LA: Investigation, Project administration, Writing – review & editing. GR: Investigation, Project administration, Writing – review & editing. FC: Investigation, Project administration, Writing – review & editing. OD: Visualization, Writing – review & editing. RU: Conceptualization, Writing – review & editing.
